# Prognostic Factors and Initial Treatment Strategies for Patients with Head Trauma and Vital Signs of Shock

**DOI:** 10.1089/neur.2024.0167

**Published:** 2025-04-18

**Authors:** Masaki Yasuda, Makoto Ohtake, Taisuke Akimoto, Masayuki Okano, Yuya Imanishi, Takafumi Kawasaki, Jun Suenaga, Katsumi Sakata, Ichiro Takeuchi, Tetsuya Yamamoto

**Affiliations:** ^1^ Department of Neurosurgery, Yokohama City University Graduate School of Medicine, Yokohama, Japan.; ^2^ Department of Emergency and Critical Care, Yokohama City University Medical Center, Yokohama, Japan.; ^3^ Department of Neurosurgery, Yokohama City University Medical Center, Yokohama, Japan.

**Keywords:** prognosis, shock, traumatic brain injury, vital signs

## Abstract

Head trauma accompanied by circulatory failure is a rare but severe condition, and few reports regarding its prognosis or initial treatment strategies have been published. We aimed to evaluate the prognostic factors and treatment strategies for patients with head trauma and vital signs of shock. We included 415 consecutive patients with head trauma (Abbreviated Injury Scale [AIS] score ≥3) who were transported to our institution from January 2017 to December 2023. These patients were divided into shock and non-shock groups. Data on their background, vital signs at presentation, trunk injury status, surgical intervention, and hematological findings were examined. A retrospective analysis was conducted with the modified Rankin Scale score after 3 months as the primary outcome. The patients’ mean age was 53.9 ± 24.4 years, 304 (73.3%) were male, 265 (63.9%) experienced severe trauma (injury severity score ≥16), 124 (29.9%) had multiple trauma (AIS score ≥3 at two or more locations), and 59 (14.2%) had accompanying vital signs of shock (shock index >1). Multivariable analysis revealed that older age (*p* < 0.0001), a lower Glasgow Coma Scale (GCS) score (*p* < 0.0001), elevated D-dimer levels (*p* = 0.0077), and pupillary abnormalities (*p* = 0.038) were independently associated with a poor prognosis in the non-shock group. In the shock group, older age (*p* = 0.0037) and neurosurgical intervention (*p* = 0.012) were independent prognostic factors. In contrast to those in the non-shock group, the GCS score and D-dimer levels were not useful prognostic factors in the shock group. The optimal cut-off age for prognosis was 64 years (area under the receiver operating characteristic curve: 0.752; sensitivity: 0.670, specificity: 0.777). The prognosis was significantly worse in the shock group when neurosurgery was required, suggesting that developing a treatment strategy aimed at more rapidly reducing intracranial pressure is essential, especially for patients under 64 years old with circulatory failure.

## Introduction

Traumatic brain injury (TBI) is a major cause of traumatic death and will likely surpass many other conditions as a leading cause of death and disability. When TBI is combined with severe trunk injuries, the prognosis worsens even further. TBI reportedly occurs in 25%–50% of cases of multiple trauma, and it poses a risk of secondary brain damage due to vital signs of shock.^1,2^ Therefore, to improve the prognosis of patients with both severe TBI and trunk injuries, comprehensive knowledge and experience are required in the field of emergency care for multiple-trauma treatment, not just in the field of neurosurgery.

Prognostic factors need to be discovered to improve our understanding of the nature and optimize treatment strategies for TBI. The most commonly reported prognostic factors for TBI are older age, a lower Glasgow Coma Scale (GCS) score, and higher D-dimer levels.^3–5^ Coagulopathy is common in patients with head trauma and affects the clinical course and prognosis. During the acute phase of trauma, coagulopathy can cause massive bleeding and worsen the prognosis of patients with severe trauma. This condition includes consumptive coagulopathy, caused by tissue injury and hypoperfusion due to circulatory failure, and hyperfibrinolytic disseminated intravascular coagulation, which is mainly caused by enhanced fibrinolytic activity, resulting in elevated D-dimer levels.^6^ In previous reports, D-dimer levels of 50 µg/ml or higher correlated with an increased risk of hematoma,^3^ and the mortality rate was 7.11 times higher in those with a D-dimer level of 50 µg/ml or higher than that in those with a D-dimer level of 15 µg/ml or lower.^7–9^ In contrast, Zhang et al. reported that a higher D-dimer level upon admission was associated with a higher risk of progressive hemorrhagic injury but not with a higher risk of a Glasgow Outcome Scale score ≤3 at 3 months,^10^ indicating that D-dimer may not be a prognostic factor for head trauma depending on the patient’s general condition or complications.

To eliminate preventable deaths associated with TBI and trunk injuries, patients with serious conditions who can be saved must be identified and treated as soon as possible. The hyperfibrinolytic state of such patients complicates the achieving of hemostasis, ultimately leading to circulatory failure, which is an important factor in their management. To our knowledge, no studies on prognostic factors for patients with TBI have been focused on those with vital signs of shock. This may be partly because vital signs of shock are very rare in cases of TBI, as most patients experience elevated blood pressure due to Cushing’s reflex, which is associated with high intracranial pressure (ICP).

In this study, we focused on patients with vital signs of shock as those most likely to be critically ill and have coagulation abnormalities among those with TBI. Yokohama City University Medical Center is a tertiary emergency facility/severe trauma center in Japan. It admits relatively many patients with severe head trauma who exhibit vital signs of shock at the time of admission. In this retrospective study, we analyzed treatment outcomes and prognostic factors for patients with head trauma and vital signs of shock, aiming to determine the optimal treatment strategies for such patients.

## Materials and Methods

### Study design and participants

The study protocol was reviewed and approved by the Institutional Review Board and Ethics Committee of Yokohama City University Medical Center (approval no. B200700036). The requirement for written informed consent was waived and individual informed consent was obtained on an opt-out basis owing to the retrospective study design, in line with the Act on the Protection of Personal Information and National Research Ethics Guideline in Japan. The research was completed in accordance with the Declaration of Helsinki as revised in 2013.

This single-center, retrospective cohort study included 415 consecutive patients aged ≥1 year with head trauma (Abbreviated Injury Scale [AIS] score ≥3) who were transported to our institution from January 2017 to December 2023. These patients were divided into the shock and non-shock groups. Age, sex, trauma history, vital signs, pupillary findings, blood test results at presentation, the shock index (SI), the injury severity score (ISS), the AIS score, the Rotterdam computed tomography (CT) score, the presence or absence of skull or pelvic fractures, the presence or absence of subdural hematoma (SDH) or epidural hematoma (EDH), neurosurgical intervention, and transarterial embolization (TAE) were examined. Prognostic analysis was conducted for the shock group, including the time from hospital arrival to a resolution of vital signs of shock (defined as an SI <1.0 or a mean arterial pressure >90 mmHg sustained for at least 15 min). In this study, an ISS ≥16 was defined as severe trauma,^11^ and an AIS score ≥3 in two or more locations was defined as multiple trauma.^12^ The SI was used for the initial assessment of hypovolemic shock, as proposed by Allgower et al. in 1967, and was defined as the heart rate divided by the systolic blood pressure. An SI >1.0 indicated hemorrhagic shock.^13^ The Rotterdam CT score is used to predict mortality based on head-CT features such as the basal cistern status, presence of a midline shift >5 mm, presence of EDH, and presence of intraventricular hemorrhage and/or subarachnoid hemorrhage, as mentioned in earlier studies.^14,15^

### Treatment protocol

When a patient with severe trauma is transported to our facility, a physiological evaluation is usually performed to identify conditions that require urgent intervention, in accordance with the Advanced Trauma Life Support course and Japan Advanced Trauma Evaluation and Care guidelines.^16,17^ Patients judged to have difficulty with airway management upon arrival were immediately intubated and ventilated in the primary care room. Circulation was managed according to guidelines of the Brain Trauma Foundation,^17^ and sufficient volume loading with extracellular fluid was performed, especially in patients with vital signs of shock. The target mean blood pressure was >90 mmHg. Red blood cells were transfused when hemoglobin was <7.0 g/dl, and if massive bleeding was suspected, blood transfusion was performed at a plasma:platelet:erythrocyte ratio of 1:1:1 according to international protocols for massive transfusion.^17,18^ Tranexamic acid was administered to almost all patients with traumatic hemorrhage. In cases in which whole-body contrast-enhanced CT revealed persistent bleeding in the trunk, resuscitative endovascular balloon occlusion of the aorta (REBOA), emergency laparotomy, or TAE was performed to stop the bleeding; if necessary, an ICP sensor was inserted to manage the cerebral perfusion pressure (CPP). Intracranial interventions were generally performed after hemodynamic stabilization. In cases requiring emergency hematoma removal in the primary care room, the procedure was performed only on patients whose hemodynamics responded to initial treatment and in whom blood pressure could be maintained through volume loading, in preparation for a sudden drop in blood pressure following ICP release. If an intracranial hemorrhage with mass effect was observed upon head CT, craniotomy was performed for hematoma evacuation and decompression.^19^ After acute treatment was completed, the patient was maintained in the intensive care unit until their condition stabilized.

### Outcome measures

The primary endpoint was the modified Rankin Scale (mRS) score at 3 months. For prognostication, patients with an mRS score of 0–2 were classified as the good prognosis group, whereas those with an mRS score of 3–6 were classified as the poor prognosis group. Each patient’s 3-month post-injury condition was evaluated via phone calls, letters, or outpatient examinations.

### Statistical analysis

To account for biases, the analysis was performed after the completion of the study period. The results are presented as means ± standard deviations (ranges) or medians (ranges) for quantitative data and as frequencies (percentages) for categorical data. For comparisons between groups, Pearson’s chi-square (or Fisher’s exact) and Wilcoxon tests were performed. The area under the receiver operating characteristic curve was used to test the predictive ability of the model. A value of 0.70–0.79 was regarded as average discrimination, 0.80–0.89 as good discrimination, and 0.90–1.00 as excellent discrimination. Statistical significance was set at *p* < 0.05. All statistical analyses were performed using JMP 15 software (SAS Institute Inc., Cary, NC, USA).

## Results

The characteristics of the 415 patients are summarized in Table 1. The mean age was 53.9 ± 24.4 (1–97) years, and 304 (73.3%) patients were male. Overall, 265 (63.9%) patients experienced severe trauma, 124 (29.9%) had multiple trauma, and 255 (61.4%) had a GCS score ≤13 at presentation. Head CT revealed that 217 patients (52.3%) had SDH and 72 (17.3%) had EDH. In total, 59 patients (14.2%) were classified as having vital signs of shock. Pelvic fractures occurred in 57 (13.7%) patients, 153 patients (36.9%) underwent neurosurgical intervention, and 34 (8.2%) underwent TAE. The mean hospital stay was 28.2 ± 29.9 (1–236) days, and the good prognosis group comprised 215 patients (51.8%). Fifty-three deaths (12.8%) occurred during hospitalization, none of which were related to the neurosurgical intervention.

**Table 1.** Characteristics and Univariate Analysis of Individual Factors Between the Non-shock and Shock Groups

Variable	Total *n* = 415	Non-shock *n* = 356	Shock *n* = 59	*p* value
Age (years), mean ± SD	53.9 ± 24.4	54.8 ± 24.3	48.3 ± 24.6	0.069
Sex (male)	304 (73.3)	267 (75.0)	37 (62.7)	**0.048**
GCS score, median (range)	9 (3–15)	10 (3–15)	8 (3–15)	0.053
Dyscoria (anisocoria + bilateral mydriasis)	83 (20.0)	68 (19.1)	15 (25.4)	0.29
Skull fracture	200 (48.2)	173 (48.6)	27 (45.8)	0.63
Subdural hematoma	217 (52.3)	185 (52.0)	32 (54.2)	0.74
Epidural hematoma	72 (17.3)	65 (18.3)	7 (11.9)	0.23
Rotterdam score, median (range)	2 (0–6)	2 (0–6)	3 (1–6)	**0.019**
Neurosurgical intervention	153 (36.9)	132 (37.1)	21 (35.6)	0.83
TAE	34 (8.2)	18 (5.1)	16 (27.1)	**<0.001**
Pelvis fracture	57 (13.7)	35 (9.8)	22 (37.3)	**<0.001**
ISS ≥16	265 (63.9)	216 (60.7)	49 (83.1)	**<0.001**
Head and neck AIS, median (range)	4 (3–6)	4 (3–6)	4 (3–5)	0.144
Two or more AIS score ≥3 sites	124 (29.9)	87 (24.4)	37 (62.7)	**<0.001**
Blood test results at the time of visit				
Lactate (mmol/L), median (range)	2.6 (0.5–100)	2.4 (0.5–100)	4.8 (0.8–99.6)	**<0.001**
pH (hydrogen ion exponent), mean ± SD	7.35 ± 0.11	7.36 ± 0.10	7.30 ± 0.14	**0.0013**
Hemoglobin (g/dL), mean ± SD	12.7 ± 2.6	12.9 ± 2.7	11.6 ± 2.0	**<0.001**
Platelet (×10000/µL), mean ± SD	218.8 ± 77.9	220.2 ± 77.9	211.2 ± 77.7	0.55
D-dimer (µg/ml), median (range)	28.25 (0.5–951.2)	24.5 (0.5–951.2)	65.8 (0.7–754.2)	**<0.001**
Fibrinogen (µg/ml), mean ± SD	254.0 ± 104.9	263.2 ± 107.0	198.4 ± 90.9	**<0.001**
Period of hospitalization (days), median (range)	20 (1–236)	18 (1–210)	33 (1–236)	0.061
mRS score 0–2 at discharge	200 (48.2)	174 (48.9)	26 (44.1)	0.49
Death at discharge	53 (12.8)	41 (11.5)	12 (20.3)	0.062

Data are presented as the frequency (%), unless otherwise indicated. Bold letters mean significant data with *p* < 0.05.

AIS, Abbreviated Injury Scale; GCS, Glasgow Coma Scale; ISS, Injury Severity Score; mRS, modified Rankin Scale; SD, standard deviation; TAE, transarterial embolization.

In 59 patients with vital signs of shock, the most common cause of circulatory failure was pelvic fracture (22 cases, 37.3%), followed by intra-abdominal injury (16, 27.1%), and 16 (27.1%) patients required TAE for hemostasis. Among the patients with shock, neurosurgical intervention was required for 21 (35.6%), all of whom underwent surgery after their circulatory dynamics had stabilized. The shock group was relatively young (48.3 ± 24.6 vs. 54.8 ± 24.3 years), with higher prevalences of severe (49/59 [83.1%] vs. 216/356 [60.7%]; *p* < 0.001) and multiple (37/59 [62.7%] vs. 87/356 [24.4%]; *p* < 0.001) trauma, higher Rotterdam scores (3 [1–6] vs. 2 [0–6]; *p* = 0.019), and higher D-dimer levels (65.8 µg/ml vs. 24.5 µg/ml; *p* < 0.001).

Multivariable analysis of prognostic factors based on the mRS score at 3 months in the non-shock group revealed that older age (*p* < 0.0001), a lower GCS score (*p* < 0.0001), elevated D-dimer level (*p* = 0.0077), and pupillary abnormalities (*p* = 0.038) were independently associated with poor prognosis (Table 2). The optimal cut-off value of D-dimer for prognosis in the non-shock group was 35 µg/ml (area under the receiver operating characteristic curve: 0.716; sensitivity: 0.561; specificity: 0.801). In the multivariable analysis of the 59 patients with shock, older age (*p* = 0.0037) and neurosurgical intervention (*p* = 0.012) were independent prognostic factors (Table 3). The optimal cut-off age for prognosis was 64 years (area under the receiver operating characteristic curve: 0.752; sensitivity: 0.670, specificity: 0.777). In both groups, CT features (presence of SDH or EDH, Rotterdam score) were not significantly associated with the prognosis. In the shock group, the duration of vital signs of shock was not associated with the prognosis (47.2 ± 28.7 vs. 44.2 ± 22.7 min; *p* = 0.707).

**Table 2.** Univariate and Multivariable Analysis of Prognostic Factors in the Non-shock Group

Variable	90 days mRS score 0–2 *n* = 182	90 days mRS score 3–6 *n* = 174	Univariate	Multivariable	
			*p* value	Odds ratio (95% CI)	*p* value
Age (years)	44.1 ± 24.2	66.0 ± 18.8	**<0.001**	1.05 (1.04–1.07)^a^	**<0.001**
Sex (male)	141 (77.5)	126 (72.4)	0.271		
GCS score, median (range)	13 (3–15)	6 (3–15)	**<0.001**	0.81 (0.74–0.87)^a^	**<0.001**
Dyscoria (anisocoria + bilateral mydriasis)	20 (11.0)	48 (27.6)	**<0.001**	2.32 (1.05–5.17)	**0.038**
Skull fracture	92 (50.5)	81 (46.6)	0.391		
Subdural hematoma	78 (42.9)	107 (61.5)	**<0.001**	1.32 (0.74–2.35)	0.339
Epidural hematoma	39 (21.4)	26 (14.9)	0.119		
Rotterdam score, median (range)	2 (0–6)	2 (0–5)	0.112		
Neurosurgical intervention	55 (30.2)	77 (44.3)	**0.0061**	1.11 (0.60–2.07)	0.751
Systolic blood pressure (mmHg), mean ± SD	138.7 ± 29.2	154.5 ± 44.4	**<0.001**	1.00 (0.99–1.01)^a^	0.558
TAE	5 (2.7)	13 (7.5)	**0.042**	2.69 (0.61–11.2)	0.190
Pelvis fracture	14 (7.7)	21 (12.1)	0.166		
ISS ≥16	103 (56.6)	113 (64.9)	0.107		
Two or more AIS score ≥3 sites	46 (25.3)	41 (23.6)	0.707		
Blood test results at the time of visit					
Lactate (mmol/L), median (range)	2 (0.6–100)	2.9 (0.5–79.7)	**<0.001**	1.01 (0.96–1.04)^a^	0.662
pH (hydrogen ion exponent), mean ± SD	7.37 ± 0.09	7.34 ± 0.11	**0.025**	1.16 (0.04–36.6)^a^	0.931
Hemoglobin (g/dL), mean ± SD	13.4 ± 2.9	12.4 ± 2.3	**0.0013**	0.96 (0.86–1.07)^a^	0.425
Platelets (×10000/µL), mean ± SD	237.9 ± 74.8	201.2 ± 76.8	**<0.001**	0.99 (0.99–1.00)^a^	0.607
D-dimer (µg/ml), median (range)	13.6 (0.5–240.3)	41.3 (1.2–951.2)	**<0.001**	1.001 (1.00–1.02)^a^	**0.0073**
Fibrinogen (µg/ml), mean ± SD	263.6 ± 92.8	262.8 ± 120.9	0.486		

Data are presented as the frequency (%) unless otherwise indicated. Bold letters mean significant data with *p* < 0.05.

^a^ Odds ratio per unit.

AIS, Abbreviated Injury Scale; CI: confidence interval; GCS, Glasgow Coma Scale; ISS, Injury Severity Score; mRS, modified Rankin Scale; SD, standard deviation; TAE, transarterial embolization.

**Table 3.** Univariate and Multivariable Analysis of Prognostic Factors in the Shock Group

Variable	90 days mRS score 0–2 *n* = 33	90 days mRS score 3–6 *n* = 26	Univariate	Multivariable	
			*p* value	Odds ratio (95% CI)	*p* value
Age (years)	40.8 ± 22.8	57.9 ± 23.9	**0.011**	1.04 (1.02–1.07)^a^	**0.0037**
Sex (male)	28 (84.8)	20 (76.9)	0.356		
GCS score, median (range)	12 (3–15)	5 (3–15)	0.071		
Dyscoria (anisocoria + bilateral mydriasis)	6 (18.2)	9 (34.6)	0.150		
Skull fracture	16 (48.5)	14 (53.8)	0.196		
Subdural hematoma	15 (45.5)	17 (65.4)	0.911		
Epidural hematoma	4 (12.1)	3 (11.5)	0.159		
Rotterdam score, median (range)	3 (1–5)	2.5 (1–6)	0.919		
Neurosurgical intervention	8 (24.2)	13 (50.0)	**0.040**	5.9 (1.58–23.9)	**0.012**
TAE	11 (33.3)	5 (19.2)	0.226		
Pelvis fracture	15 (45.5)	7 (26.9)	0.144		
ISS ≥16	26 (78.8)	23 (88.5)	0.488		
Two or more AIS score ≥3 sites	21 (63.6)	16 (61.5)	0.869		
Shock duration (min), mean ± SD	47.2 ± 28.7	44.2 ± 22.7	0.707		
Blood test results at the time of visit					
Lactate (mmol/L), median (range)	4.25 (0.8–99.6)	4.85 (0.9–23)	0.809		
pH (hydrogen ion exponent), mean ± SD	7.32 ± 0.13	7.27 ± 0.16	0.217		
Hemoglobin (g/dL), mean ± SD	12.0 ± 2.2	11.3 ± 1.8	0.133		
Platelets (×10000/µL), mean ± SD	223.8 ± 81.5	195.2 ± 70.9	0.123		
D-dimer (µg/ml), median (range)	64.75 (0.7–581.5)	69.65 (5.1–754.2)	0.506		
Fibrinogen (µg/ml), mean ± SD	210.6 ± 102.7	181.5 ± 70.4	0.457		

Data are presented as the frequency (%) unless otherwise indicated. Bold letters mean significant data with *p* < 0.05.

^a^ Odds ratio per unit.

AIS, Abbreviated Injury Scale; CI: confidence interval; GCS, Glasgow Coma Scale; ISS, Injury Severity Score; mRS, modified Rankin Scale; SD, standard deviation; TAE, transarterial embolization.

## Discussion

Regarding the results of the analysis of the non-shock group, older age, a lower GCS score, and elevated D-dimer levels were independent prognostic factors. These results were consistent with those of previous reports on prognostic factors in single trauma of the head.^3,20,21^ Pupillary abnormalities were also significant prognostic factors, suggesting that intracranial findings are strongly associated with a poor prognosis. An analysis of the Japan Neurotrauma Data Bank revealed that the D-dimer was a significant prognostic factor in single injuries of the head, with a cut-off value of 27 µg/ml associated with a worse Glasgow Outcome Scale score at 6 months.^8,21^ This result was almost the same as the cut-off value of 35 µg/ml calculated in this study, indicating that the non-shock group in this study did not significantly differ from their population. Additionally, EDH, which is often reported as a favorable prognostic factor, was not identified as such in this study. This is likely due to the fact that this study was limited to a population with an AIS score ≥3 and was a single-center study conducted at a high-level trauma center, where mild cases were rare.

Approximately 3% of patients with head trauma reportedly have a systolic blood pressure <90 mmHg;^22^ however, to our knowledge, no reports of prognostic analyses of patients with both head trauma and vital signs of shock have been published. In our analysis of patients with TBI, age was an independent prognostic factor in both groups, and patients under 64 years had a better prognosis with optimal treatment. Thus, for patients aged <64 years with head trauma and vital signs of shock, aggressive therapeutic intervention is recommended. Additionally, the duration of vital signs of shock was not significantly associated with the prognosis. In contrast with the non-shock group, a lower GCS score and elevated D-dimer levels were not significant prognostic factors in the shock group, whereas neurosurgical intervention was a significant independent predictor of poor outcome. One potential reason why the D-dimer level was a strong independent prognostic factor in the non-shock group but not in the shock group is that most patients with vital signs of shock have a trunk injury, which results in early D-dimer elevation, especially in cases of chest or pelvic trauma, which may mask D-dimer elevation due to head trauma.^23^ Bruijns et al. reported a significantly higher 48-hour mortality rate for patients with an SI × age ≥55 if they had vital signs of shock.^24^ In addition, a reversed SI × GCS score ≤14 is reportedly associated with a sevenfold increased risk of death,^25^ indicating that the absence of D-dimer as a mortality-related factor is characteristic of shock cases. Another possible reason why a lower GCS score was not a prognostic factor in the shock group is that many cases of impaired consciousness in the shock group were associated with hemodynamic deterioration, in which case rapid hemostasis could have improved the level of consciousness. In contrast with the non-shock group, the shock group had a significantly worse prognosis when neurosurgical intervention was required. This may be owing to the fact that in cases with ICP levels high enough to require surgical intervention, CPP is markedly decreased in association with low blood pressure, resulting in early cerebral ischemia, suggesting that rapid decongestion of ICP is important, especially in patients with vital signs of shock.^26,27^ This is consistent with previous research showing that head injury determines the functional prognosis in patients with multiple trauma.^28^ In summary, our results suggest that early recovery from vital signs of shock and rapid resolution of ICP are important, especially for patients with head trauma accompanied by circulatory failure.

**FIG. 1.**
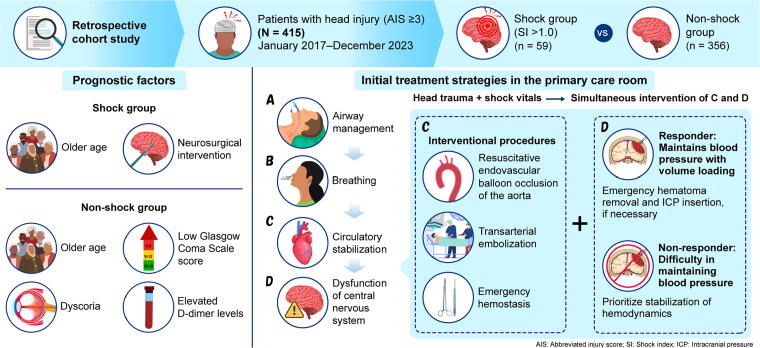
Summary of this study and initial treatment flow for patients with severe head trauma and vital signs of shock in the primary care room.

Our results led us to focus on much earlier neurosurgical intervention for patients with severe head trauma and vital signs of shock. Importantly, these results were not influenced by CT features such as EDH, SDH, or the Rotterdam score. Figure 1 presents a summary of the initial treatment strategies aimed at earlier neurosurgical intervention in the primary care room in this study. When a patient with vital signs of shock has both trunk and head injuries, we first attempt to stabilize their circulatory system as quickly as possible with extracellular fluids, blood transfusion, and circulatory agonists; when the patient’s hemodynamics respond to initial treatment, emergency perforation for hematoma evacuation and ICP monitoring is performed, simultaneously if necessary. Thereafter, hemostatic procedures, such as laparotomy, REBOA, and TAE, are performed with ICP monitoring in the primary care room. Once the patient’s hemodynamic status is stabilized, craniotomy is performed for hematoma evacuation and decompression after they are moved to the operating room. However, if the patient’s hemodynamics do not respond to initial treatment, hemostasis should be prioritized, and craniotomy should be considered after the patient’s circulation is stabilized, as a sudden drop in blood pressure with the release of ICP from an emergency perforation may lead to cardiopulmonary arrest. In the CRASH-2 study of patients with trauma and severe bleeding,^29^ and in the CRASH-3 study of patients with TBI,^30^ tranexamic acid administered within 3 h of injury was reported to improve patient outcomes. We were not able to compare patients receiving tranexamic acid with untreated patients, because almost all patients with traumatic hemorrhage are treated with tranexamic acid at our hospital. The relative risk of TBI-related death with the administration of tranexamic acid was reported as 0.78 (95% confidence interval: 0.64–0.95) for patients with mild to moderate TBI (GCS score ≥9), indicating that administration of tranexamic acid is recommended for patients with head trauma accompanied by hemorrhagic shock.

## Limitations

This study had certain limitations. Because of the single-center, tertiary-care setting, we could not avoid selection bias, as manifested in the patient characteristics. Owing to the small number of shock cases and the clinical differences in general conditions between the two groups, propensity-score matching could not be performed, leaving the possibility of confounding factors. The SI was used to define vital signs of shock, as specified in the study design phase. Although the SI is a widely used index in the emergency setting, defining shock as pulse rate/systolic blood pressure >1 might have led to the exclusion of patients with neurogenic shock,^31^ such as those who tend to develop bradycardia. Although the SI is a valid index because hemorrhage is the most common cause of shock in trauma cases at our institution, approximately 4.4% of patients have bradycardic shock, such as those with trauma-associated cervical cord injury. Nevertheless, we believe that the results of this study offer valuable insights into the treatment strategy for patients with severe head trauma and circulatory failure who can be saved, although such cases are rare. Future multicenter, prospective studies are needed to provide a high level of evidence.

## Conclusions

In patients with TBI and vital signs of shock, high D-dimer levels and low GCS scores were not significant prognostic factors. In contrast, neurosurgical intervention was an independent predictor of poor prognosis in such patients, whereas it was not a prognostic factor in the non-shock group. For patients with ICP levels high enough to require neurosurgical intervention, earlier hemodynamic stabilization and rapid reduction of intracranial hypertension are important, especially for patients under 64 years of age with vital signs of shock.

## Transparency, Rigor, and Reproducibility

For transparency, all experimental protocols and methodologies are described in detail in the Materials and Methods section. Any deviations from standard protocols are noted. All data generated and analyzed during this study are available from the corresponding author upon reasonable request. The raw data supporting the findings of this study have been saved in our university database.

To ensure the robustness of our findings, we adhered to the highest standards of experimental rigor. Statistical analyses were conducted using established methods, and all p-values, confidence intervals, and effect sizes were reported.

To facilitate the reproducibility of our work, we have provided a comprehensive description of all materials and methods used, including the specific details of the software.

## Data Availability

All data generated and analyzed during this study are available from the corresponding author upon reasonable request.

## Abbreviations Used

AIS: Abbreviated Injury Scale
CPP: cerebral perfusion pressure
CT: computed tomography
EDH: epidural hematoma
GCS: Glasgow Coma Scale
ICP: intracranial pressure
ISS: injury severity score
mRS: modified Rankin Scale
REBOA: resuscitative endovascular balloon occlusion of the aorta
SDH: subdural hematoma
SI: shock index
TAE: transarterial embolization
TBI: Traumatic brain injury
